# Supplementary figures and images for: Environmentally friendly polymers are used to enhance the water retention capacity of waste residue and the potential for vegetation growth
Source: PLoS One. 2025 Nov 14;20(11):e0332470. doi: 10.1371/journal.pone.0332470 (PMC12617928; doi:10.1371/journal.pone.0332470)

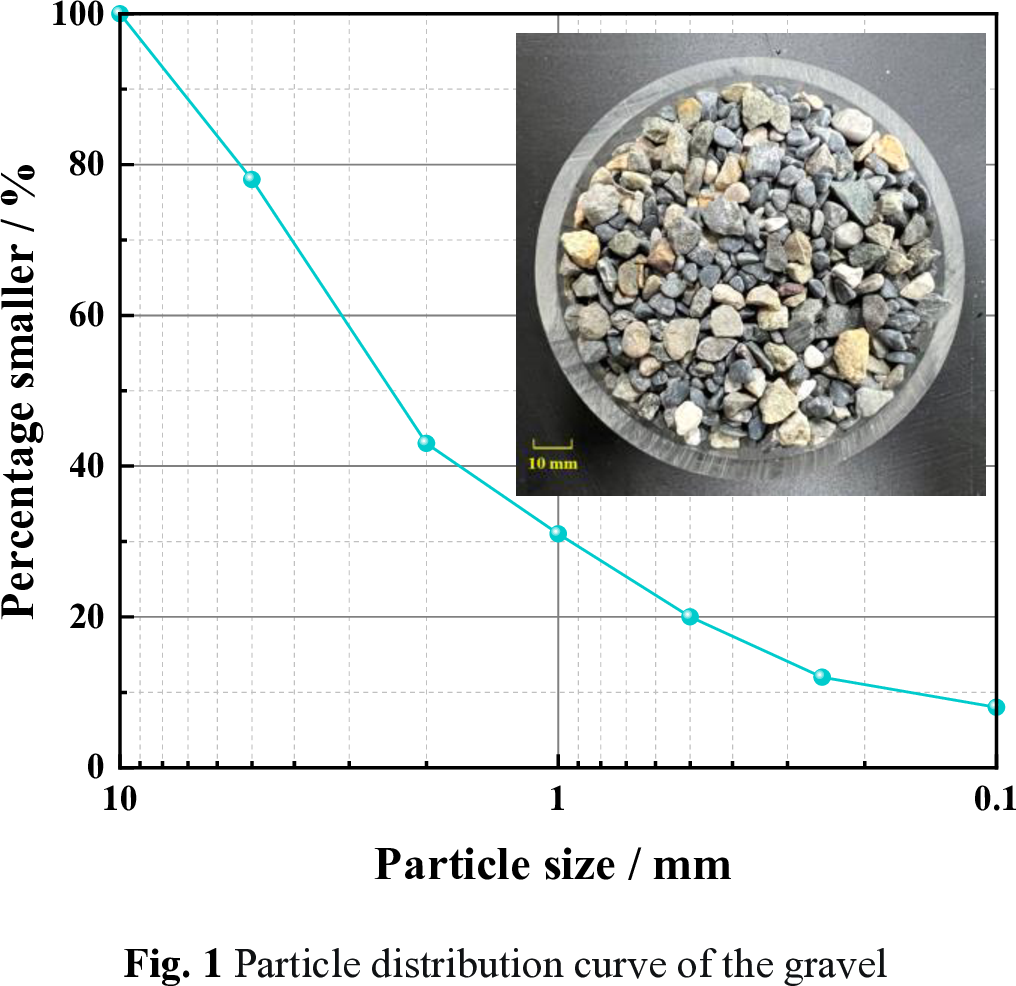

Supplement: S1 File — (ZIP) [file pone.0332470.s001.zip › Figures/Fig.1.tif]

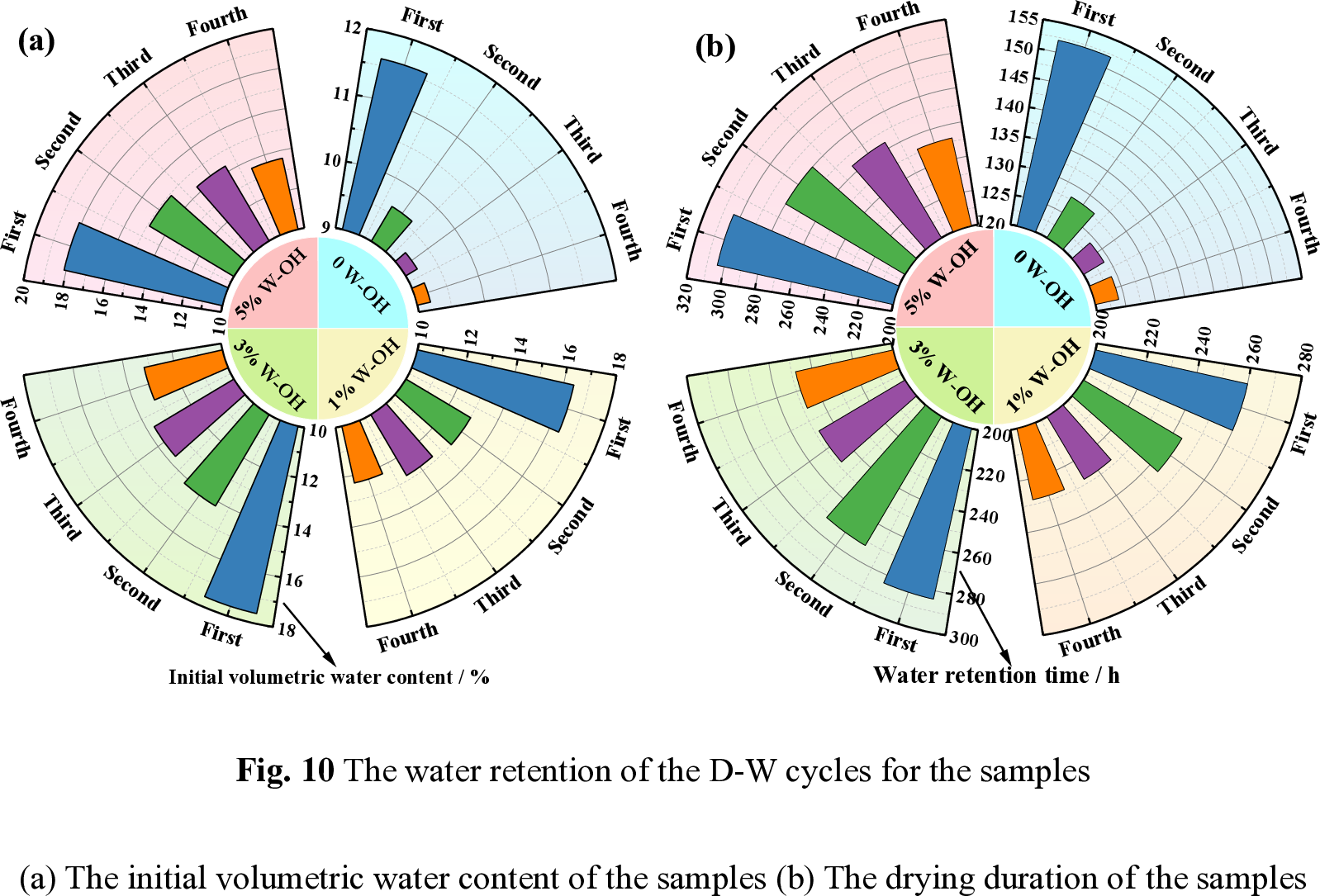

Supplement: S1 File — (ZIP) [file pone.0332470.s001.zip › Figures/Fig.10.tif]

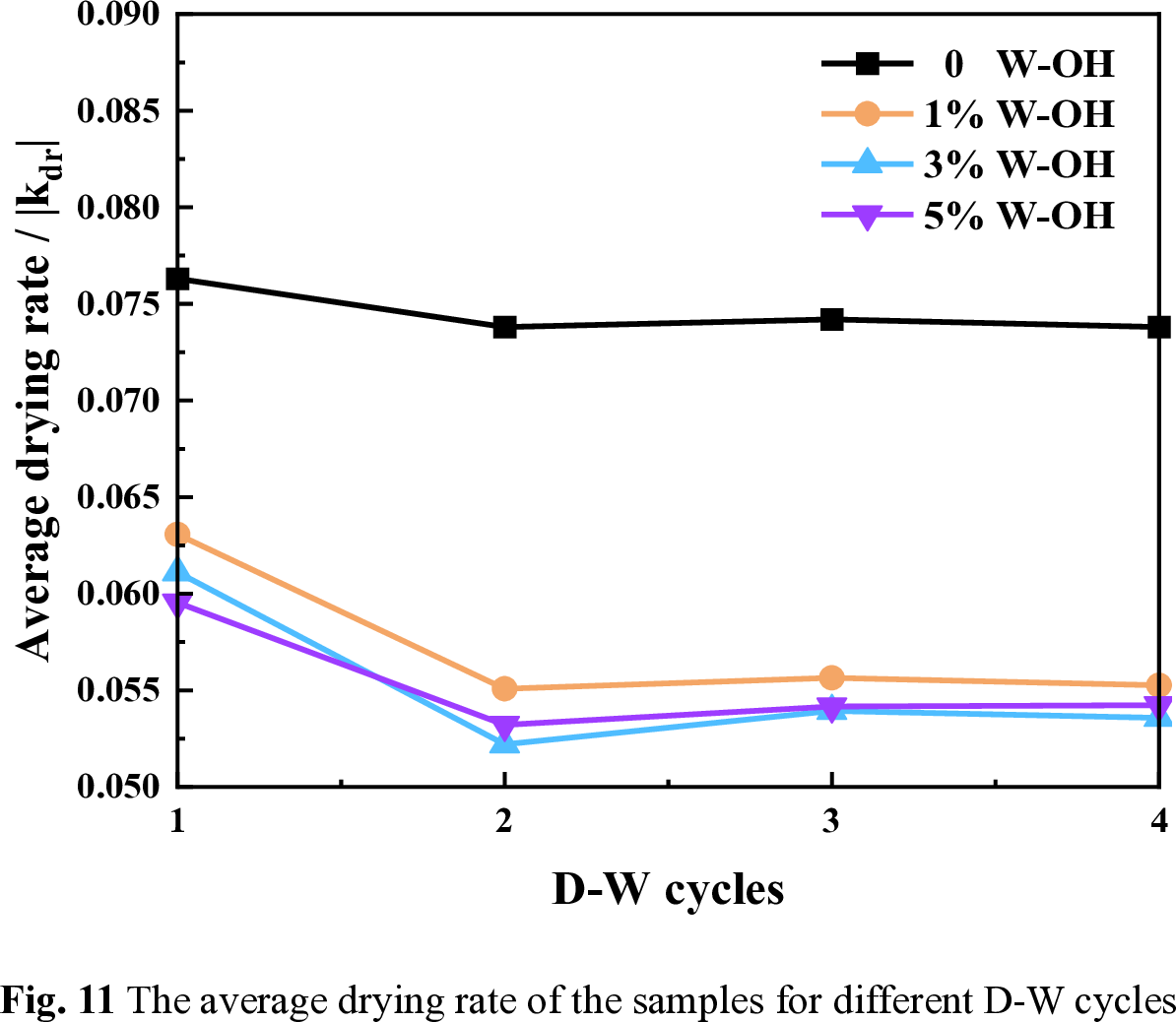

Supplement: S1 File — (ZIP) [file pone.0332470.s001.zip › Figures/Fig.11.tif]

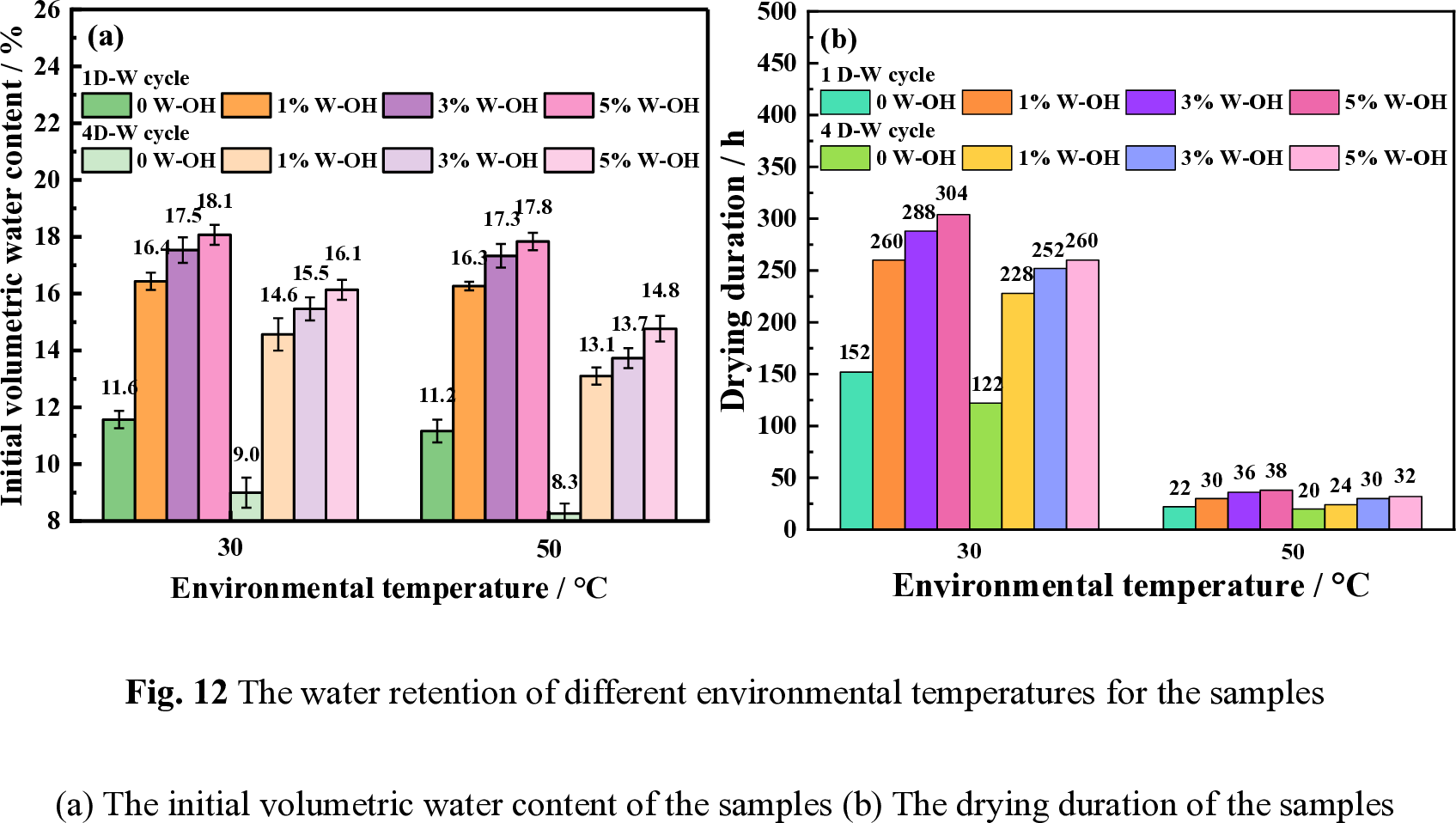

Supplement: S1 File — (ZIP) [file pone.0332470.s001.zip › Figures/Fig.12.tif]

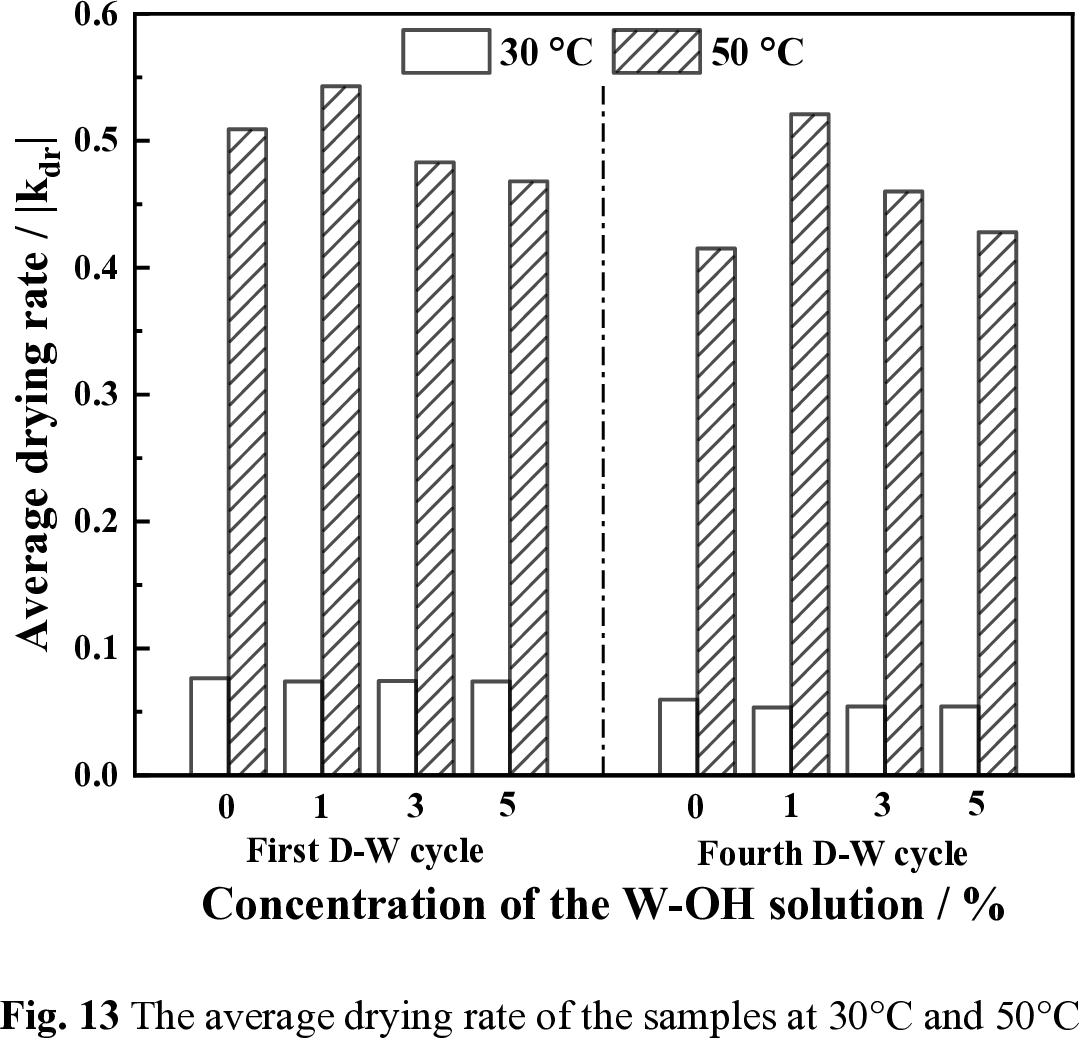

Supplement: S1 File — (ZIP) [file pone.0332470.s001.zip › Figures/Fig.13.tif]

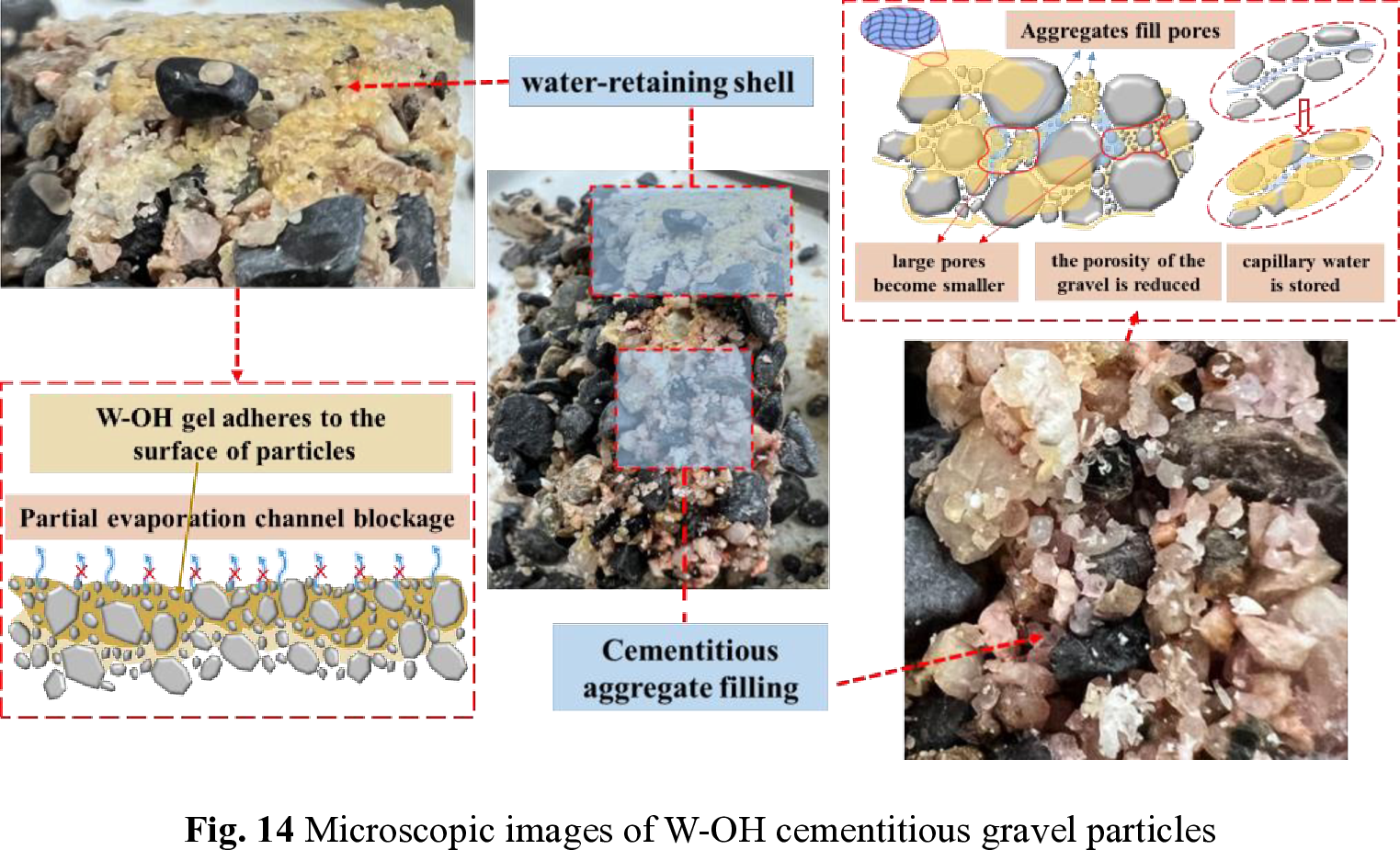

Supplement: S1 File — (ZIP) [file pone.0332470.s001.zip › Figures/Fig.14.tif]

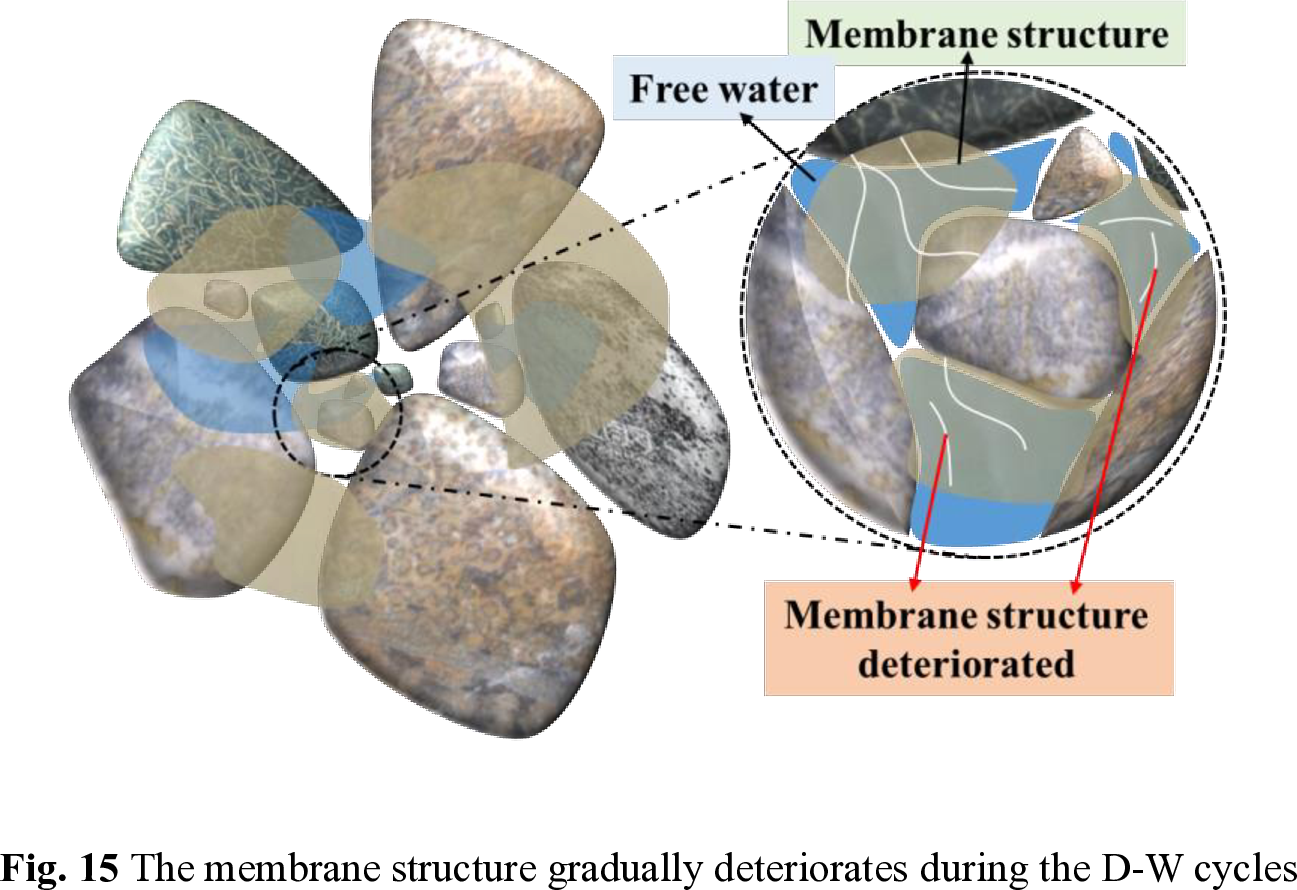

Supplement: S1 File — (ZIP) [file pone.0332470.s001.zip › Figures/Fig.15.tif]

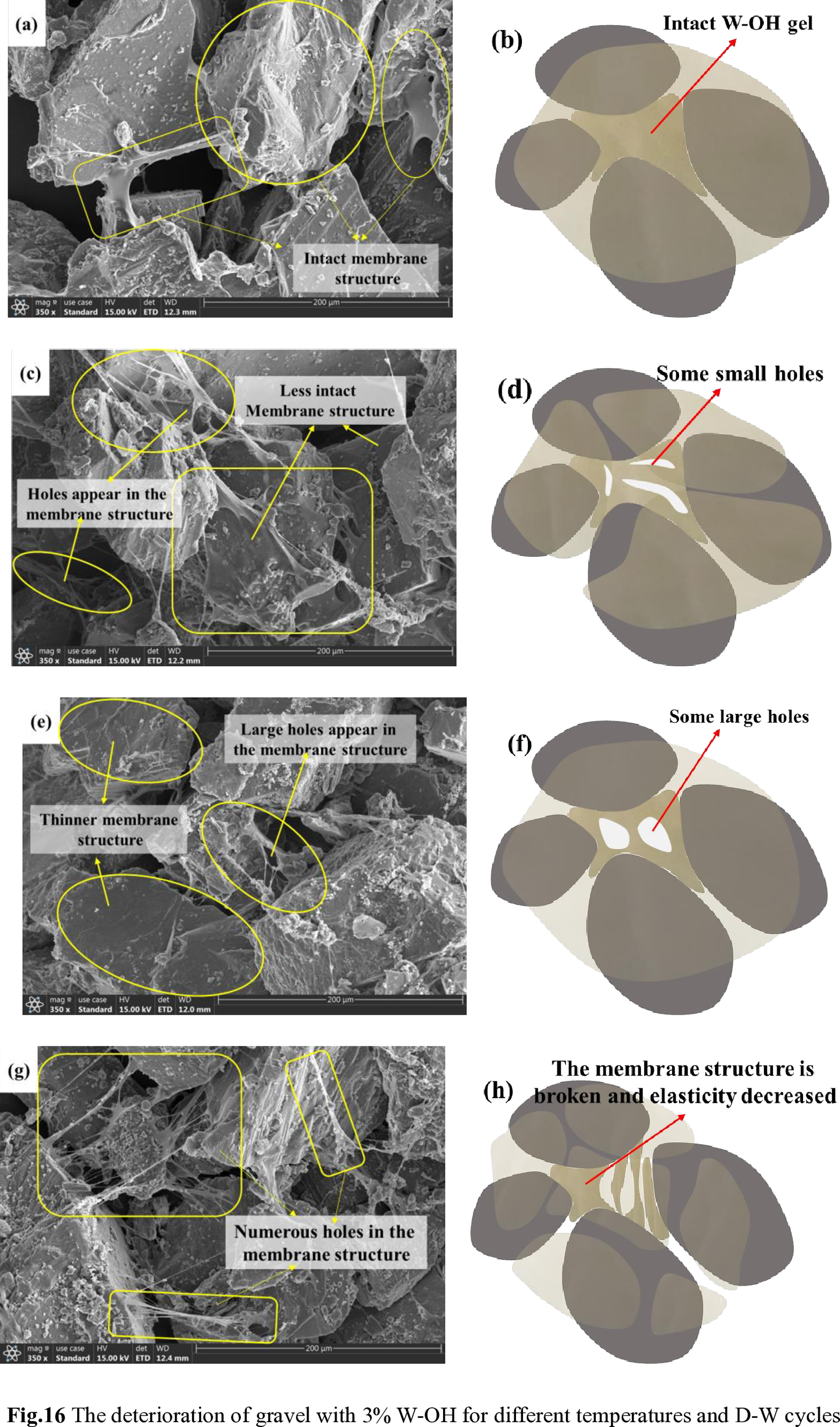

Supplement: S1 File — (ZIP) [file pone.0332470.s001.zip › Figures/Fig.16.tif]

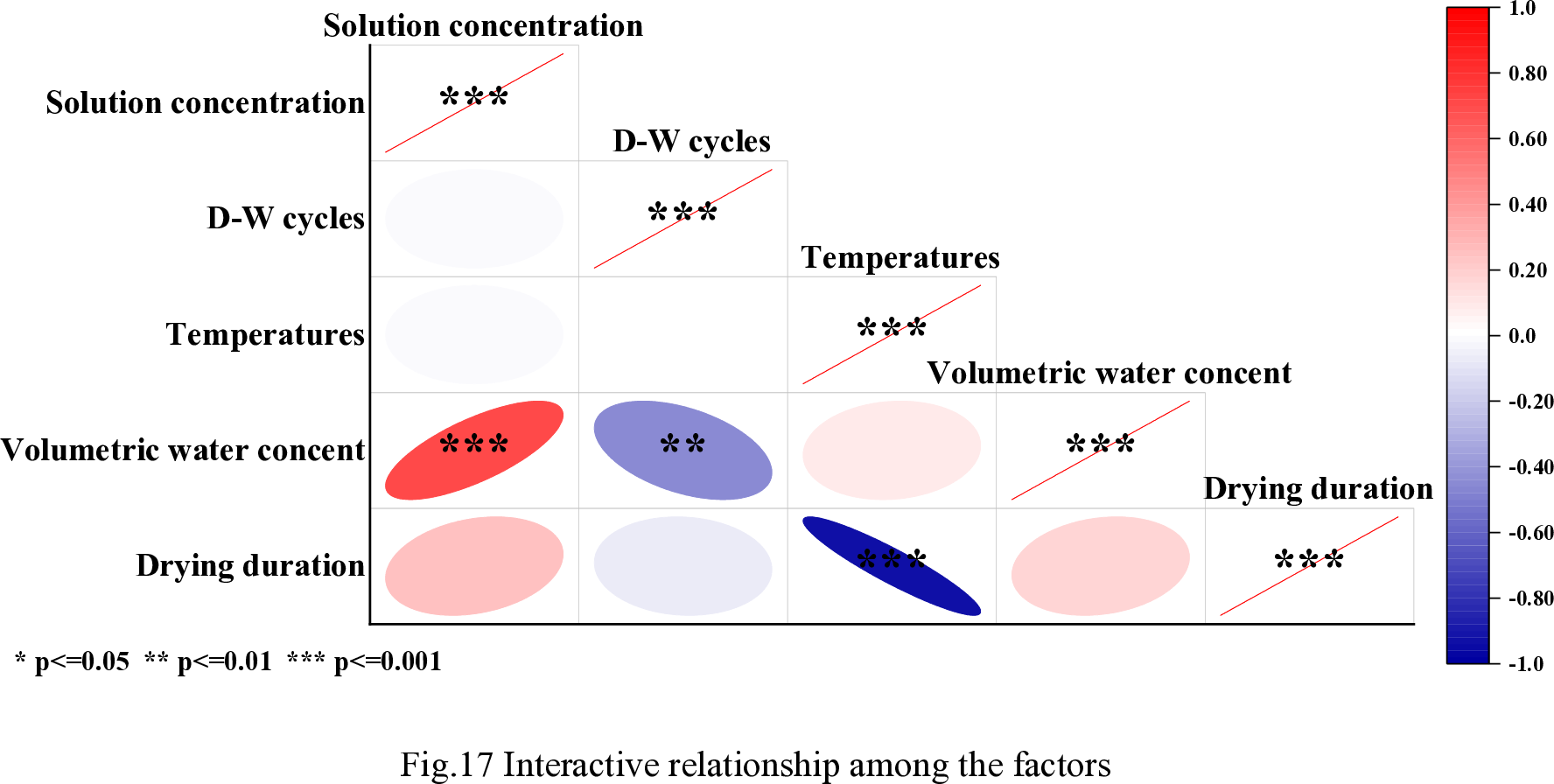

Supplement: S1 File — (ZIP) [file pone.0332470.s001.zip › Figures/Fig.17.tif]

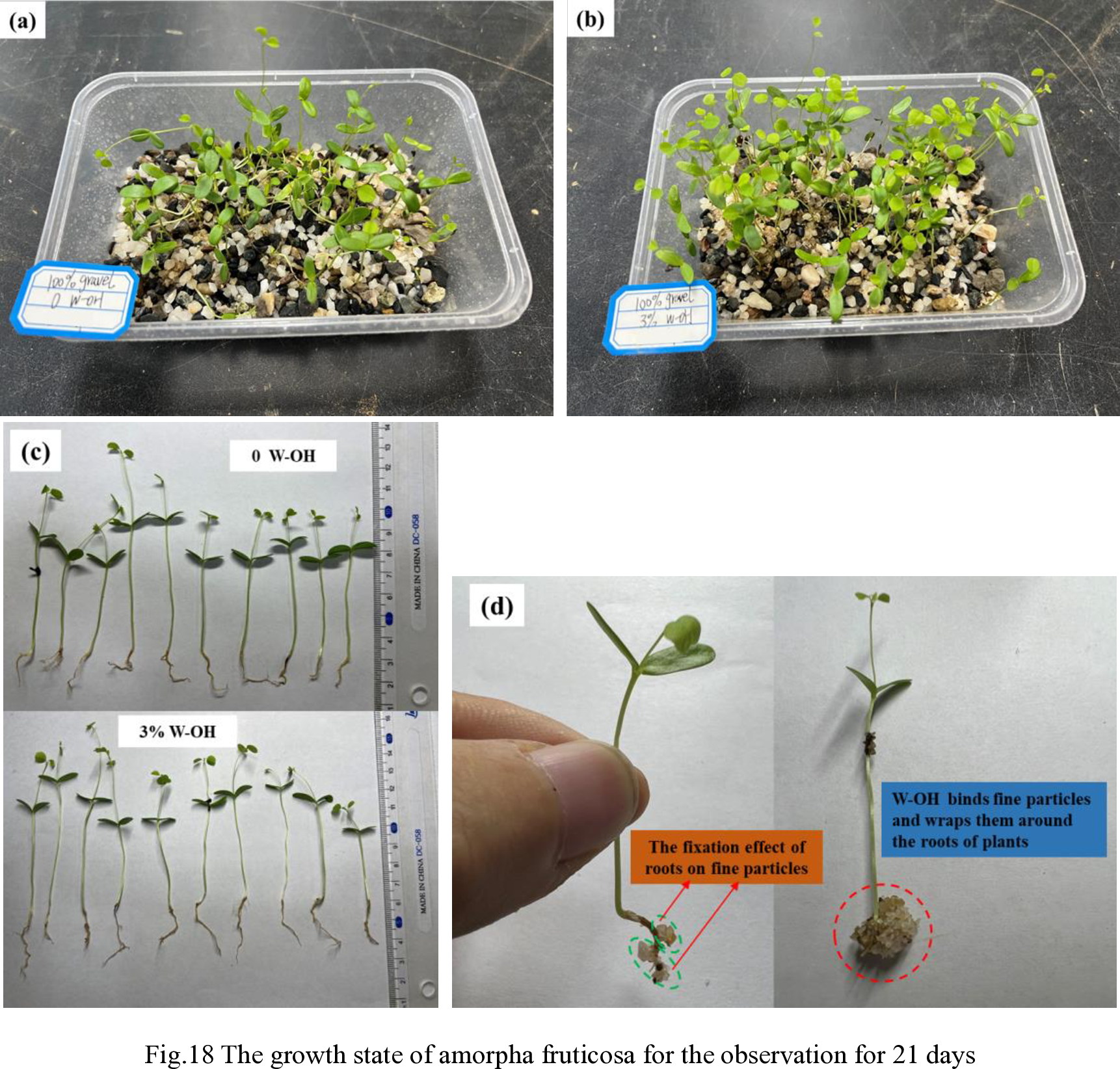

Supplement: S1 File — (ZIP) [file pone.0332470.s001.zip › Figures/Fig.18.tif]

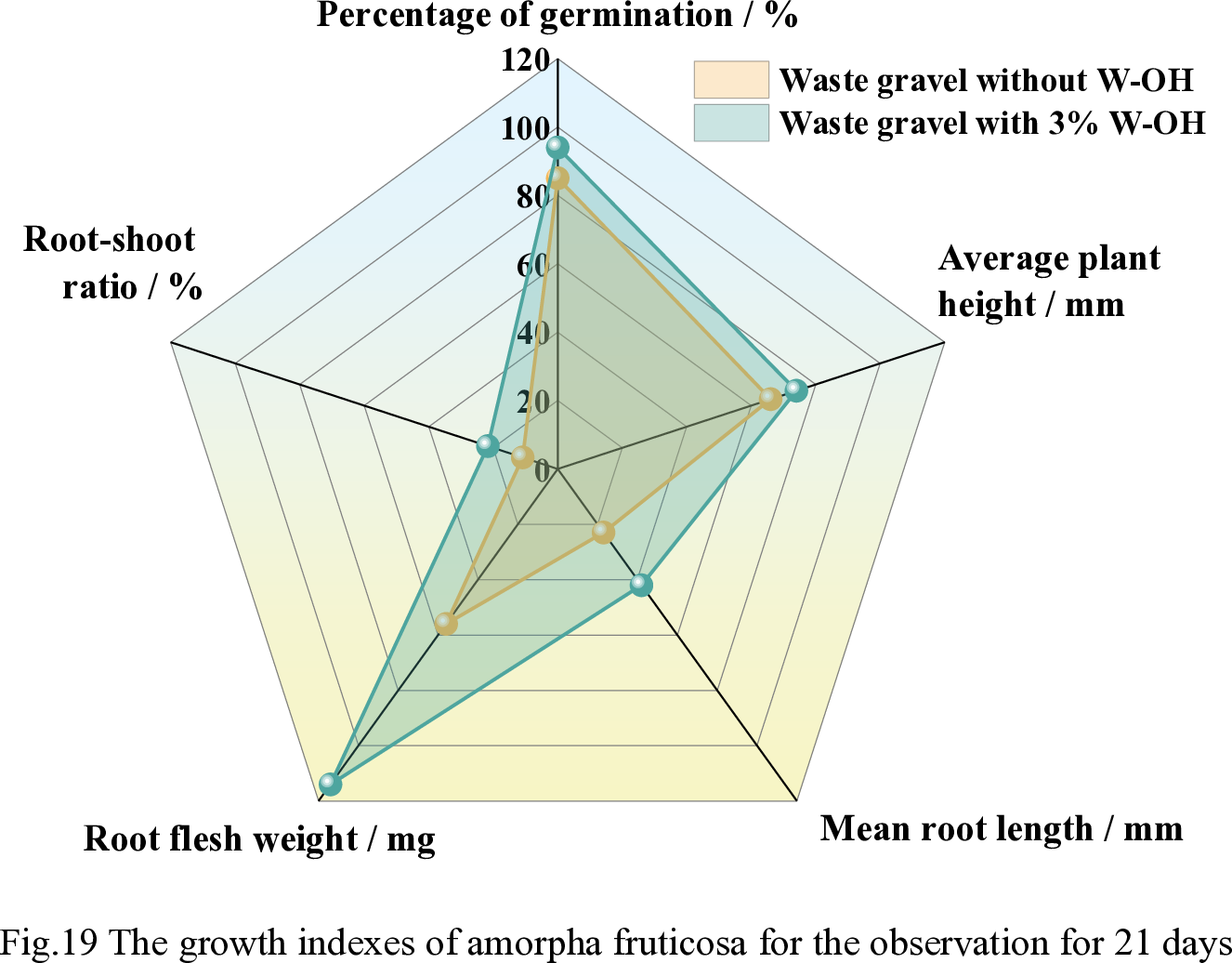

Supplement: S1 File — (ZIP) [file pone.0332470.s001.zip › Figures/Fig.19.tif]

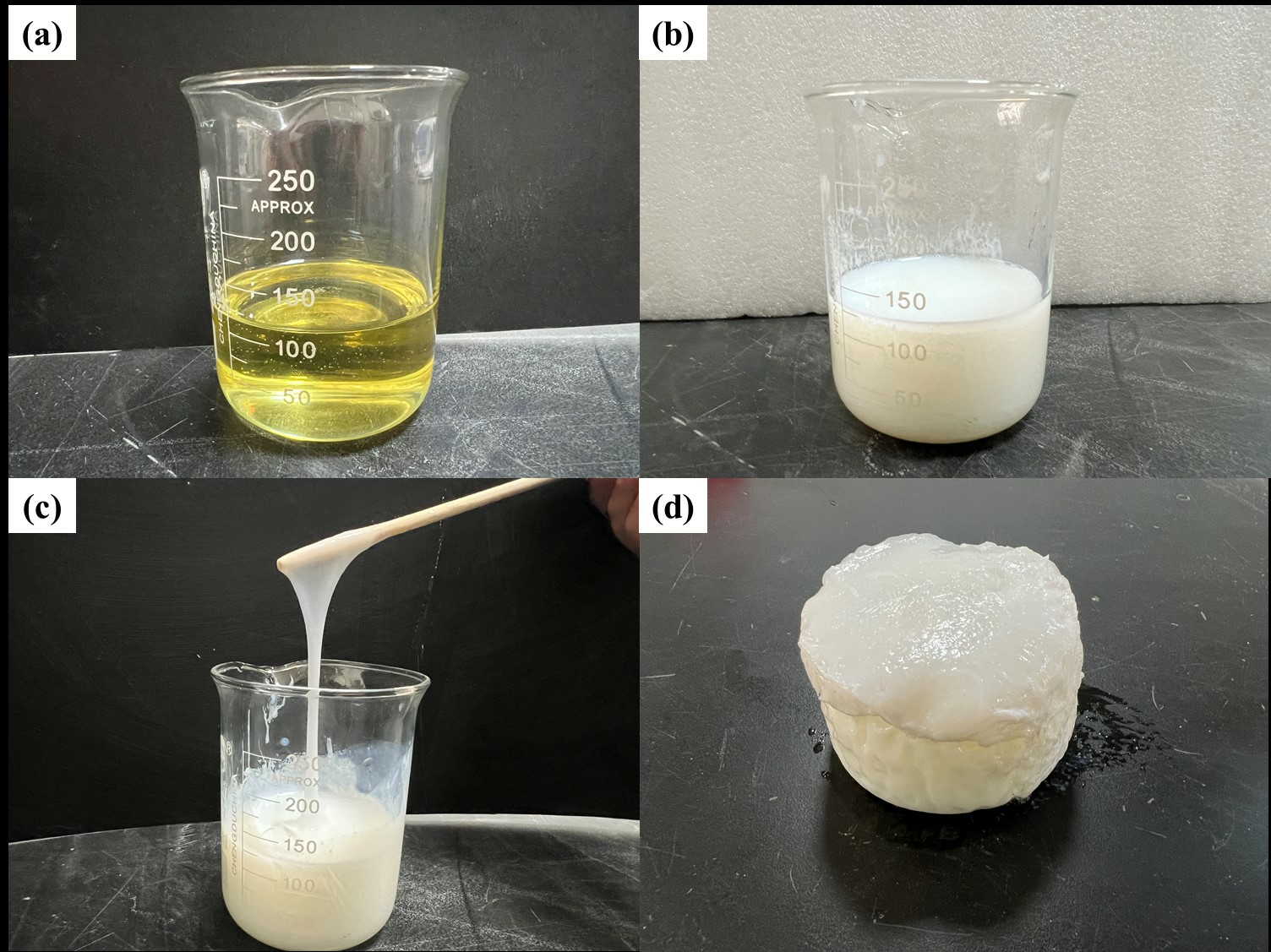

Supplement: S1 File — (ZIP) [file pone.0332470.s001.zip › Figures/Fig.2.tif]

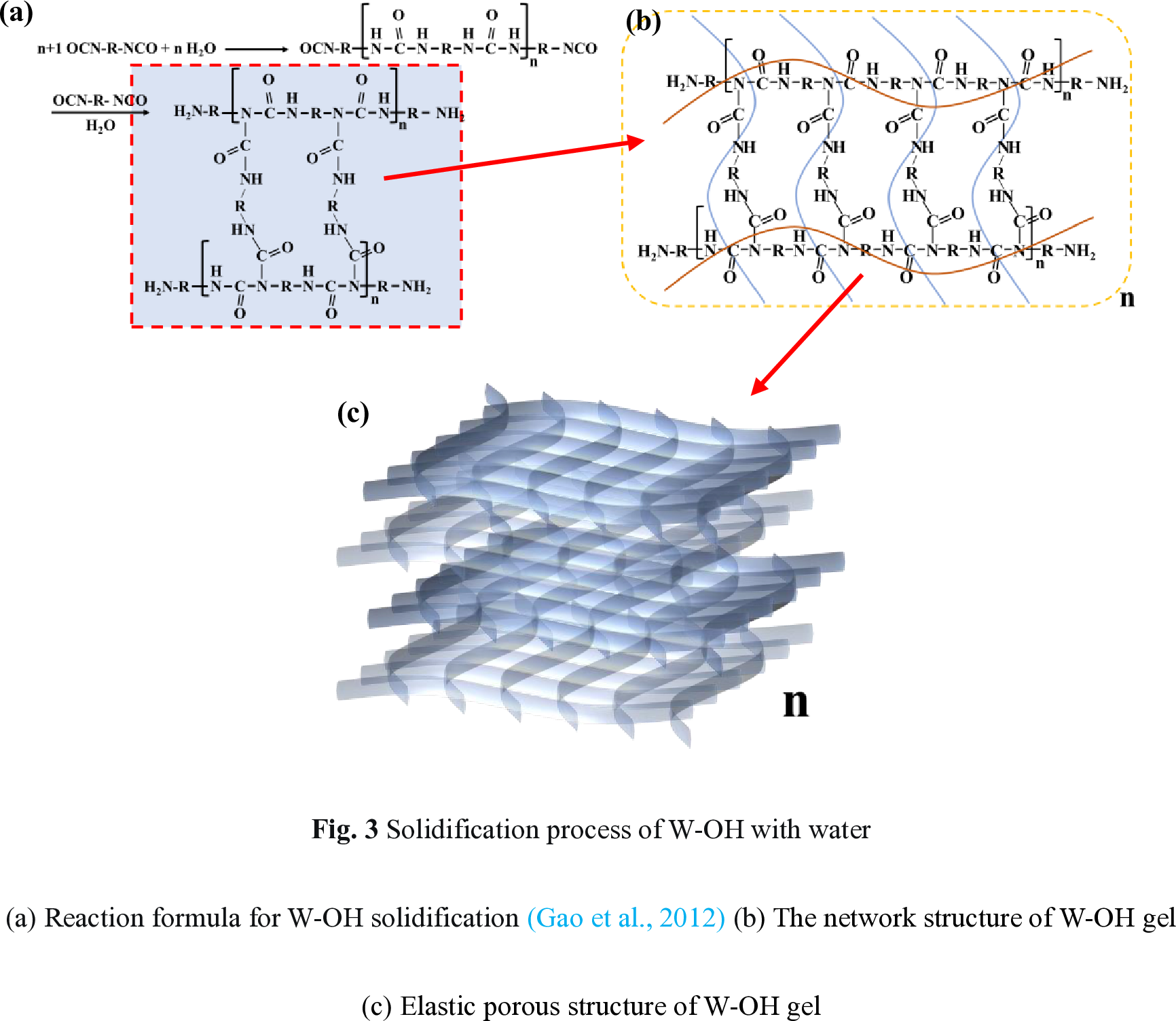

Supplement: S1 File — (ZIP) [file pone.0332470.s001.zip › Figures/Fig.3.tif]

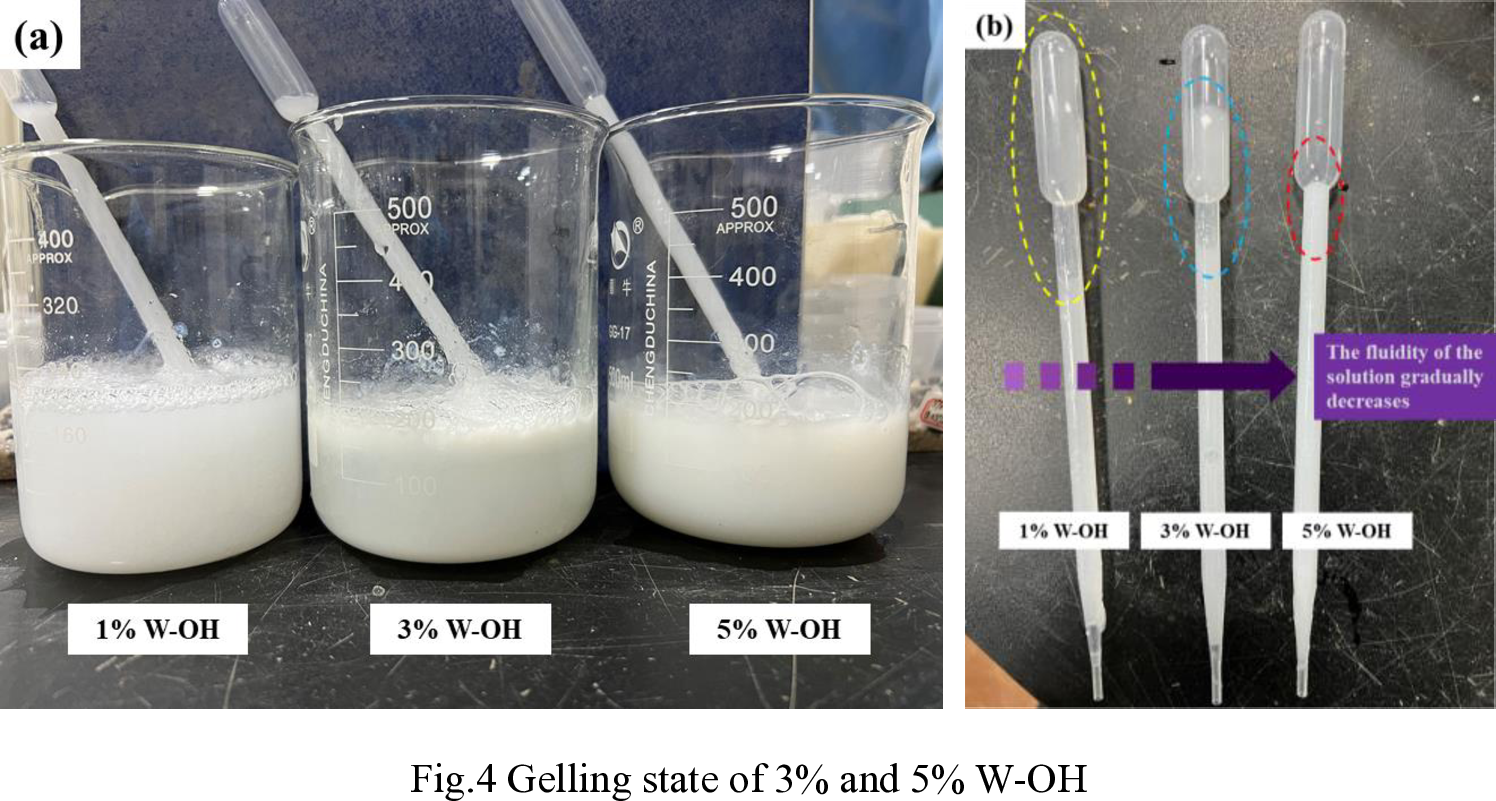

Supplement: S1 File — (ZIP) [file pone.0332470.s001.zip › Figures/Fig.4.tif]

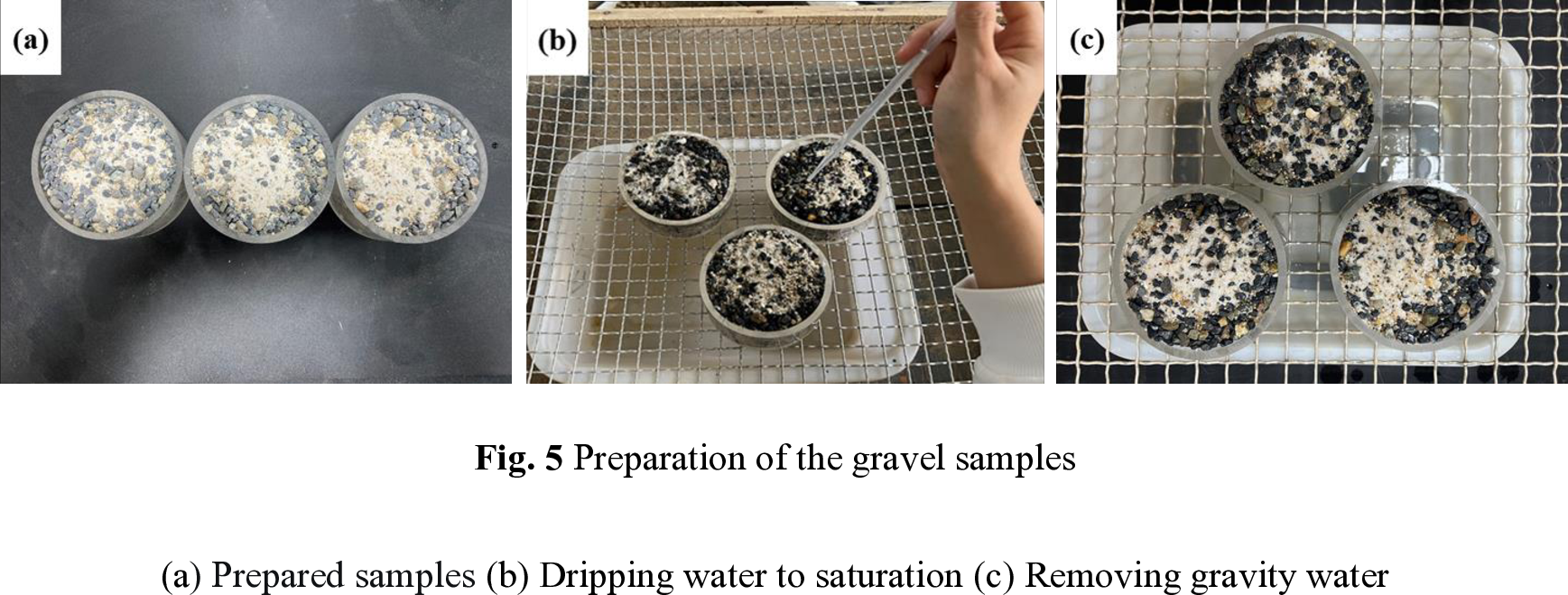

Supplement: S1 File — (ZIP) [file pone.0332470.s001.zip › Figures/Fig.5.tif]

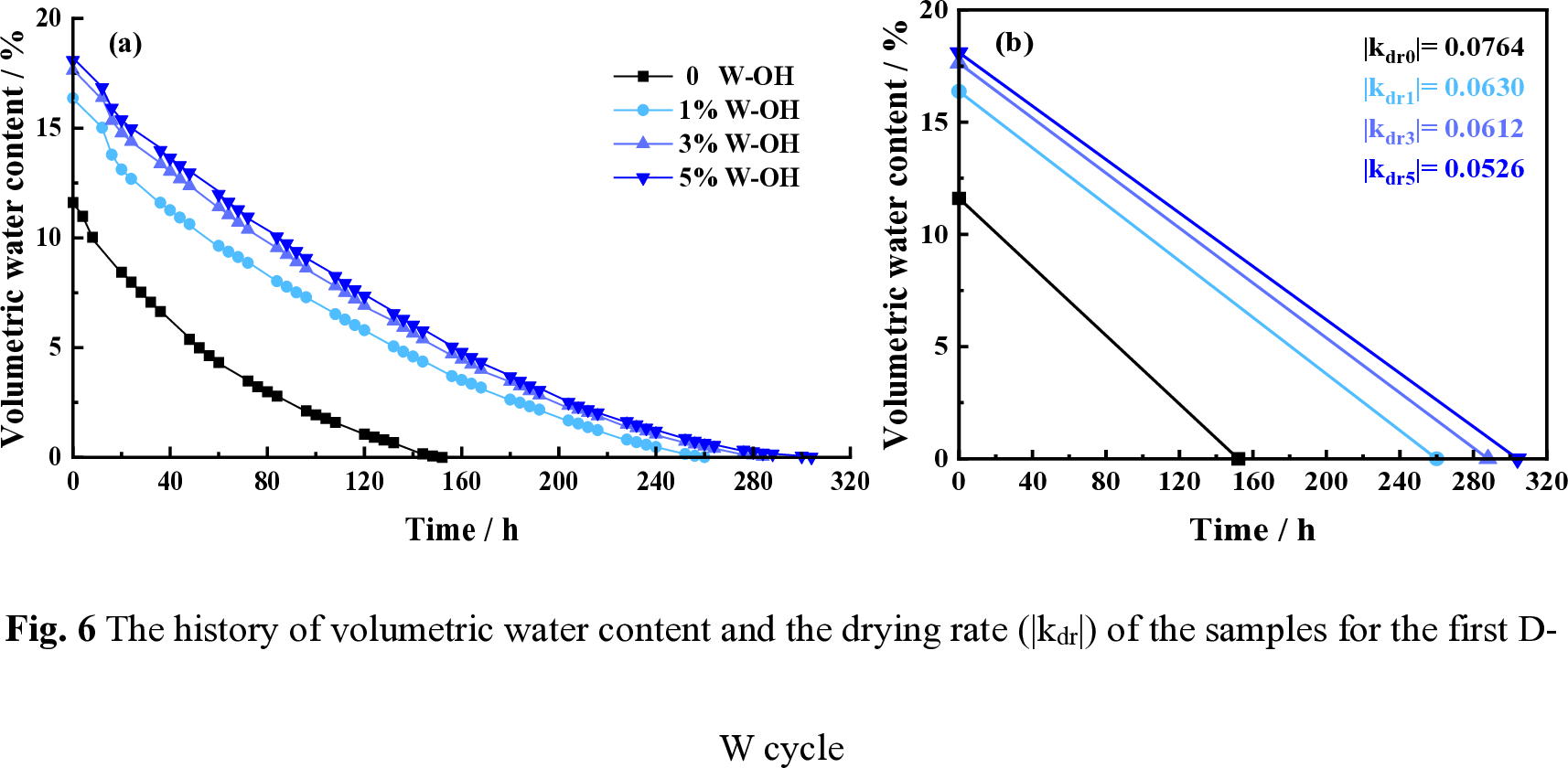

Supplement: S1 File — (ZIP) [file pone.0332470.s001.zip › Figures/Fig.6.tif]

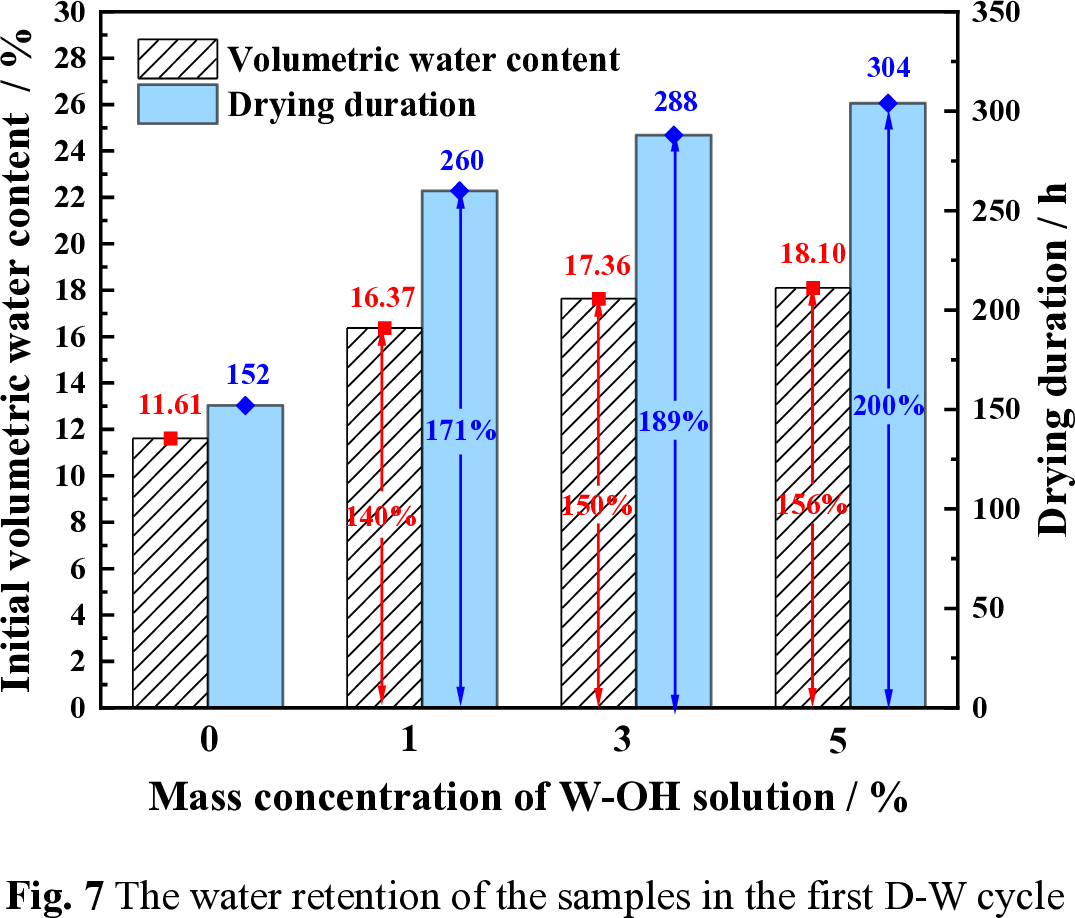

Supplement: S1 File — (ZIP) [file pone.0332470.s001.zip › Figures/Fig.7.tif]

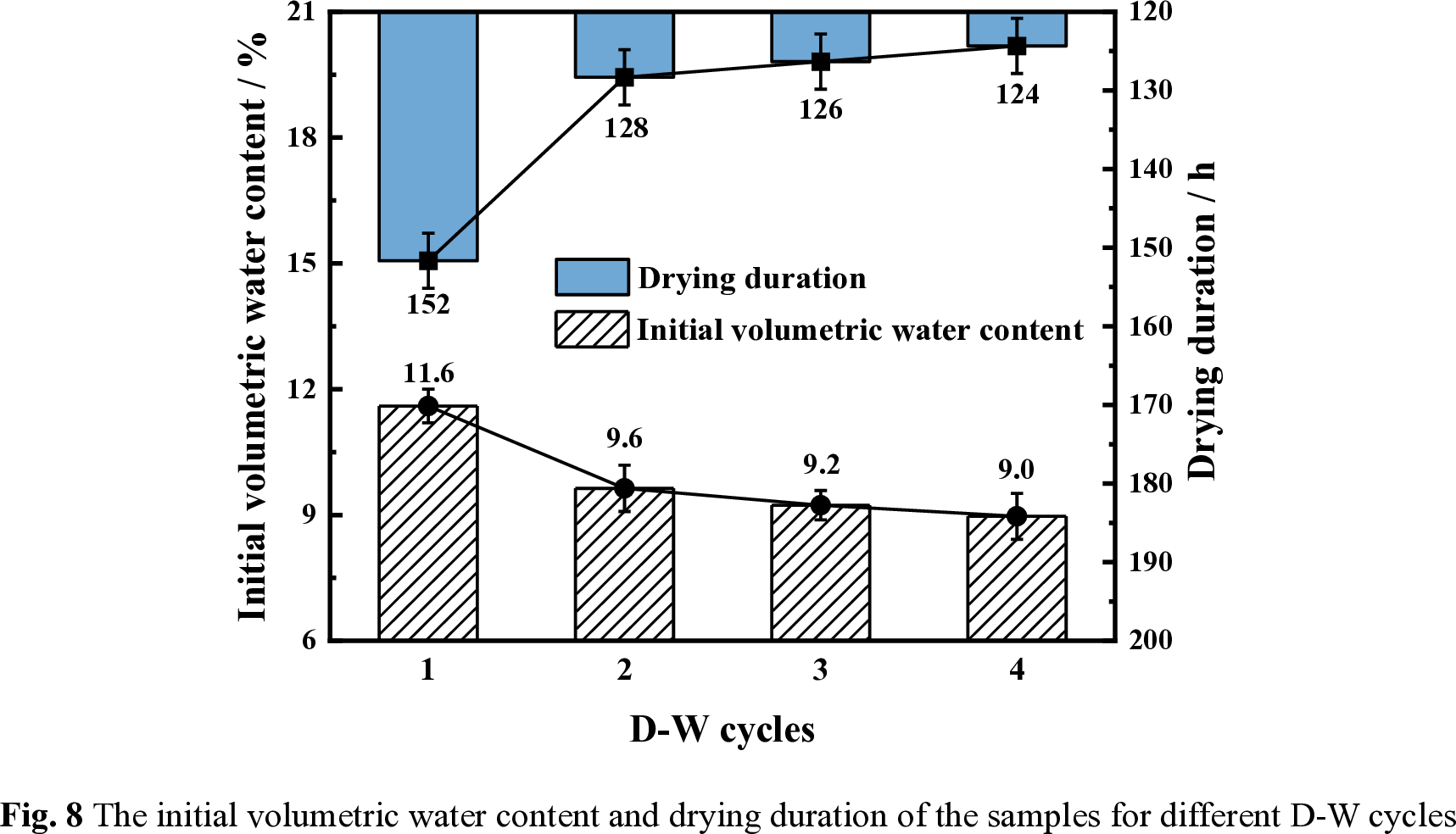

Supplement: S1 File — (ZIP) [file pone.0332470.s001.zip › Figures/Fig.8.tif]

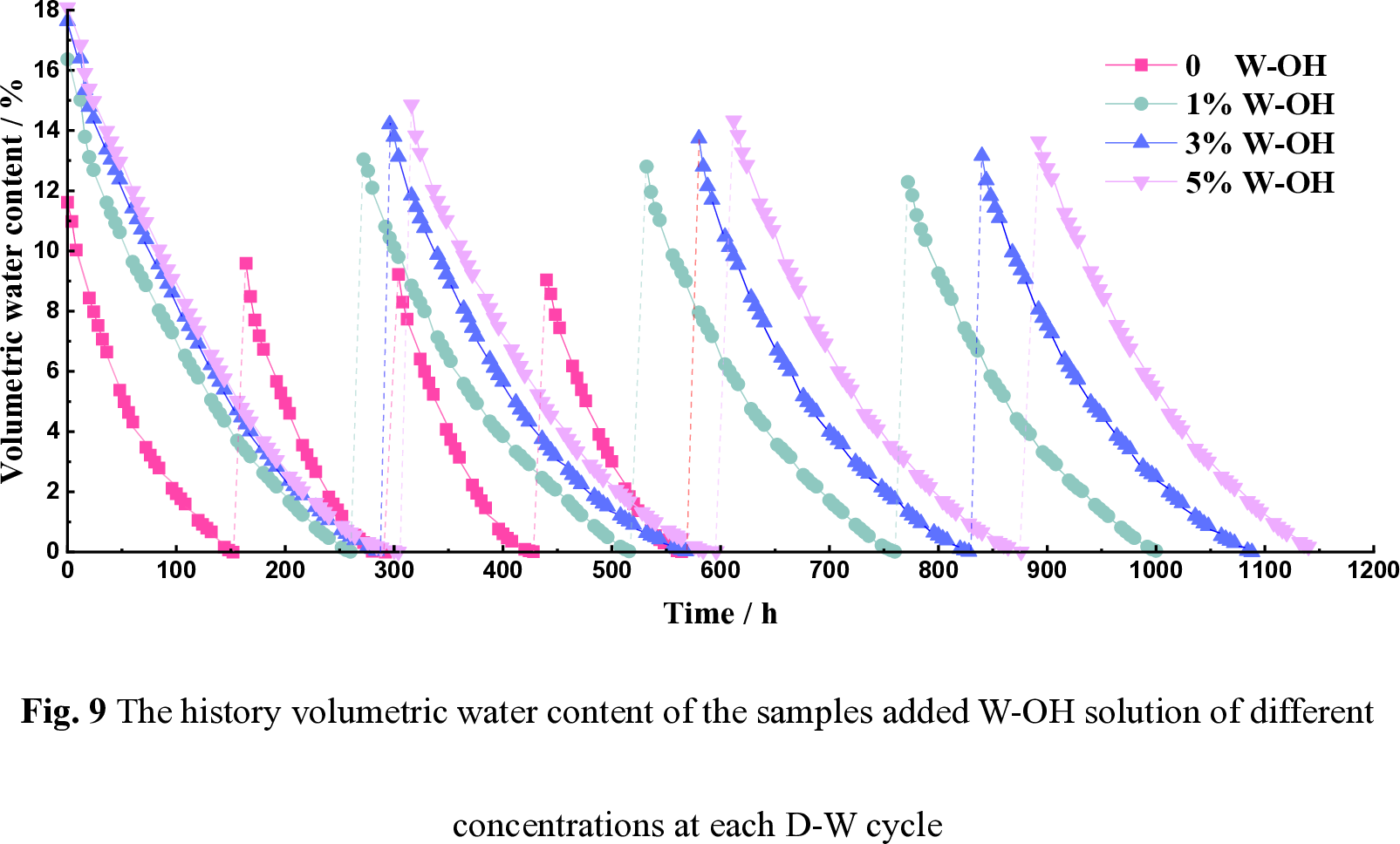

Supplement: S1 File — (ZIP) [file pone.0332470.s001.zip › Figures/Fig.9.tif]
