# Supplementary material for: Environmentally friendly polymers are used to enhance the water retention capacity of waste residue and the potential for vegetation growth
Source: PLoS One. 2025 Nov 14;20(11):e0332470. doi: 10.1371/journal.pone.0332470 (PMC12617928; doi:10.1371/journal.pone.0332470)
Supplement: S2 File — (DOC) [file pone.0332470.s002.doc]

**Table 1** The physical property parameters of gravel

| Parameter | *C_u_* | *C_c_* | *ρ*_max_ (g/cm^3^) | *G_s_* | *k* (cm/s) |
| --- | --- | --- | --- | --- | --- |
| Value | 17.65 | 1.96 | 1.85 | 2.69 | 5.2×10^-3^ |

**Table 2** Test scheme

| Test Group | Concentration of W-OH solution (%) | D-W cycles | Environmental temperature (°C) |
| --- | --- | --- | --- |
| 1 | 1, 3, 5 | 1 | 30 |
| 2 | 1, 3, 5 | 1, 2, 3, 4 | 30 |
| 3 | 1, 3, 5 | 1, 4 | 30, 50 |
